# Does a referral from home to hospital affect satisfaction with childbirth? A cross-national comparison

**DOI:** 10.1186/1472-6963-7-109

**Published:** 2007-07-12

**Authors:** Wendy Christiaens, Anneleen Gouwy, Piet Bracke

**Affiliations:** 1 Department of Sociology, Ghent University, Ghent, Belgium

## Abstract

**Background:**

The Belgian and Dutch societies present many similarities but differ with regard to the organisation of maternity care. The Dutch way of giving birth is well known for its high percentage of home births and its low medical intervention rate. In contrast, home births in Belgium are uncommon and the medical model is taken for granted. Dutch and Belgian maternity care systems are compared with regard to the influence of being referred to specialist care during pregnancy or intrapartum while planning for a home birth. We expect that a referral will result in lower satisfaction with childbirth, especially in Belgium.

**Methods:**

Two questionnaires were filled out by 605 women, one at 30 weeks of pregnancy and one within the first two weeks after childbirth, either at home or in a hospital. Of these, 563 questionnaires were usable for analysis. Women were invited to participate in the study by independent midwives and obstetricians during antenatal visits in 2004–2005. Satisfaction with childbirth was measured by the Mackey Satisfaction with Childbirth Rating Scale, which takes into account the multidimensional nature of the concept.

**Results:**

Belgian women are more satisfied than Dutch women and home births are more satisfying than hospital births. Women who are referred to the hospital while planning for a home birth are less satisfied than women who planned to give birth in hospital and did. A referral has a greater negative impact on satisfaction for Dutch women.

**Conclusion:**

There is no reason to believe Dutch women receive hospital care of lesser quality than Belgian women in case of a referral. Belgian and Dutch attach different meaning to being referred, resulting in a different evaluation of childbirth. In the Dutch maternity care system home births lead to higher satisfaction, but once a referral to the hospital is necessary satisfaction drops and ends up lower than satisfaction with hospital births that were planned in advance. We need to understand more about referral processes and how women experience them.

## Background

Since the Netherlands is characterised by a unique system encouraging home births, referrals to specialist care are a typically Dutch phenomenon. Recent research found that Dutch women are less satisfied with childbirth than Belgian women [1]. Transfer to the hospital when planning for a home birth could be one of the explanations for this finding. Research on the impact of being referred to specialist care while planning for a home birth is rare, and seldom cross-national. It was our purpose to examine the impact of referrals on childbirth satisfaction in two similar countries. No other region more closely resembles the Dutch society than Belgium does. However, the two societies differ with regard to the organisation of health care in general and maternity care in particular.

### Maternity care in Belgium and the Netherlands

The Dutch government encourages home births by directing women expecting a normal birth into primary care [2]. In case of difficulties during pregnancy or labour, women are referred to the hospital [3]. If pregnancy and labour take a normal course, women can give birth at home, accompanied by a midwife and/or general practitioner or they may choose to have a short stay under supervision of the same primary caregivers [4]. In the latter case childbirth takes place in a birth clinic or, more often, in a hospital (short stay). Normal course of pregnancy and labour is defined by the VIL (Verloskundige Indiatielijst) list of obstetric indications [5], which defines the conditions that require a referral from primary to secondary care. Dutch women expecting a normal birth are not free to choose specialist care. A drawback of the Dutch birth system is the high referral rate: although 70% of Dutch women start with antenatal care in primary care, only 30% actually have a home birth [6]. As Reuwer and Bruinse [7] note one third of all planned home deliveries end up in hospital. For nulliparous women this percentage is as high as 50%. The authors [7] use the high referral rate to criticise the Dutch maternity care system for the lack of continuity of care, which is due to the work overload of the Dutch midwives and nurses.

Generally, maternity care is more women-centred in the Netherlands [7]. It is close to the ideal type of the midwifery model [8] or the humanistic approach described by Davis-Floyd [9]. This ideal type is characterised by e.g., the conceptualisation of birth as a normal physiological process. Risk selection is one of the key ideas of the Dutch system. The midwifery model emphasises observation instead of intervention, the private instead of the public sphere, and has an individual psycho-social approach instead of a statistical biological focus [8].

In Belgium there is no formal boundary between primary and secondary care. Women do not need a preauthorisation to gain access to specialist care. In consequence the majority of Belgian women go straight to an obstetrician for antenatal care. To most Belgian women the hospital is the obvious and safest place to give birth [10]. This is reflected in a percentage of hospital births that is as high as approximately 99% [11]. Maternity care is hierarchically organised and highly standardised. Medical intervention rates are high in comparison to the Netherlands. Corresponding to the ideology of the bio-medical [8] or technocratic [9] model, birth is considered normal only in retrospect.

### Referral from home to hospital in Belgium and the Netherlands

The Belgian and Dutch systems differ especially in case of referral to specialist care. In the Netherlands the community-based midwife is allowed to continue care in the hospital unless specialist care is necessary [4]. Thus, antenatal-to-intrapartum continuity as well as intrapartum continuity is mostly guaranteed, unless interventions are necessary and the obstetrician has to take over. Wiegers et al. [3] found that Dutch women who wanted to give birth at home but were transferred to hospital were as positive about the birth and the attendance of the midwife as the women who wanted to give birth in hospital. Most of the time Belgian independent midwives cannot accompany their client to the hospital. Because of the low number of women planning for a home birth, most hospitals do not have special arrangements with independent midwives for women who are referred to the hospital. So referred women are handed over to the hospital staff. Hence being referred usually implies that antenatal-to-intrapartum continuity will be lost. However, Green et al. [12] found that being cared for by one carer during labour and delivery (intrapartum continuity) seems to be more important than being cared for by a known carer. In consequence the importance of lack of antenatal-to-intrapartum continuity should not be exaggerated. Being referred from one place to another, however, leads to a gap between expectations and reality. Literature concerning satisfaction, either consumer or patient satisfaction, refers to the discrepancy between expectations and experiences as a major cause of dissatisfaction [13-15]. Research shows that women whose expectations about childbirth were met, are more satisfied [16-18]. The discrepancy theory emphasises the evaluative aspect of satisfaction [19]. If expectations are met, the corresponding values and beliefs are affirmed. If not, disappointment may bring about dissatisfaction.

This study was designed to assess the well-being of Dutch and Belgian women before and after childbirth. In this paper we assess the influence of the discrepancy between expected and actual place of delivery on satisfaction with childbirth. A Dutch study [3] showed that being transferred from one place to another (during labour) does not influence satisfaction with childbirth. Can we confirm this finding for the Netherlands, as well as for Belgium? Belgium and the Netherlands are characterised by different birth practices and ideology, resulting in diverging care trajectories. Because of the striking discontinuity of care(r) in a case of referral in the Belgian system, we expect women to be more disappointed, hence less satisfied, than their northern neighbours.

## Methods

### Selection of method

With this study we focused on comparing childbirth expectations and experiences between four groups of women: Belgian and Dutch women with a hospital or home birth. To contact as many women as possible in a short period of time, a survey by two questionnaires–one at 30 weeks of pregnancy and one within two weeks postpartum–was considered to be appropriate. From the time the invitation to participate was issued to the completion of the last questionnaire, five to eight months passed. Since the data collection was not simultaneously organised in each hospital/midwifery practice, one year–from September 2004 to September 2005-was necessary to gather the data. At 30 weeks of pregnancy, 827 women filled out the same questionnaire; 605 of those women also participated in the study in the first two weeks after delivery and completed a second questionnaire.

### Measurement

Satisfaction was measured in the second questionnaire by the Mackey Childbirth Satisfaction Rating Scale, which consists of 6 subdimensions–general satisfaction (3 items), and satisfaction with self (9 items), baby (3 items), nurse (9 items), physician (8 items), and partner (2 items)–thus reflecting the multidimensional nature of the concept. We did not assess satisfaction with physician-related aspects of birth because women with a home birth did not see a physician. The scale was designed by M. Mackey and P. Goodman who examined multiple factors for childbirth satisfaction [16]. We translated and pilot-tested the scale for Belgian and Dutch women. A linguistic specialist translated the instrument into Dutch (for Belgian as well as Dutch women). A copy of the instrument is available from the first author. The sample Goodman et al. [16] used was limited to low-risk postpartum women with uneventful vaginal deliveries, whereas our sample extends the scope to women with instrument deliveries. Respondents indicate their degree of satisfaction with each item on a 5-point Likert scale. Internal consistency reliability coefficients for this study (total scale, 0.94; self, 0.84; baby, 0.74; midwife, 0.96; partner, 0.85; and, general, 0.71) are similar to those established by Goodman et al. [16] (total scale, 0.94; self, 0.90; baby, 0.70; midwife, 0.97; partner, 0.97; and, general, 0.93). For each subscale, means are calculated.

We asked for the intended place of birth in the antenatal questionnaire using the following question: *Where would you like to give birth? *This variable consists of two broad categories, the *home *versus the *hospital *as intended place of birth. In the postpartum questionnaire we asked for the actual place of delivery, retaining the same two categories. Women planning for a birth in a birth clinic are considered primary care clients, because a birth clinic is a substitution for the home and is not considered a medically sophisticated environment. Planning for a short stay is coded as a hospital birth, notwithstanding that in the Netherlands this is considered to be primary care. In Belgium a short stay proceeds the same way as other secondary care deliveries. Moreover we merged short stays with the hospital births' category, because in both environments medical expertise and technology are nearby in case of emergency. By comparing the intended and the actual place of birth we constructed four groups of respondents: women who planned to give birth at home and did, women who planned to give birth in hospital and did, women who planned to give birth at home, but ended up in hospital, and women who experienced other kinds of discrepancy between planned and actual place of birth (e.g., hospital to home, short stay to hospital, hospital to short stay, etc.). Country is the second independent variable in our model. It consists of two categories, Belgium and the Netherlands.

Control variables are age, level of education (0 = no higher education; 1 = higher education), and parity (0 = nulliparous; 1 = multiparous). Finally, we included method of delivery in the analysis, which consists of two categories: vaginal deliveries (0 = without interventions) versus births involving medical intervention, such as forceps, vacuum extraction or caesarean section (1 = with interventions). Women giving birth in a clinical setting, including women who have been referred, are more likely to experience a medical intervention. By taking method of delivery into account we want to make sure that the effect on satisfaction of being referred cannot be reduced to the effect of intervening during birth.

### Population and sample

Our study was conducted in Ghent and Tilburg, two comparable cities in the Belgian and Dutch regions respectively. To enhance the readability of the paper we will refer to Belgium and the Netherlands, and the Belgians and the Dutch.

In both cities all hospitals were asked to participate in the study. In Ghent there are four hospitals, of which three agreed to participate. We have no reason to believe that the population of the missing hospital differs from the population of the participating hospitals. In Tilburg both hospitals agreed to cooperate. We needed to oversample the home deliveries, since there are more hospital than home births in both countries. In Tilburg we contacted six midwifery practices to reach enough women planning a delivery in primary care. Because Ghent does not count enough midwifery practices to attain the same number of home births, we contacted 21 midwifery practices spread out over Flanders, the Dutch-speaking, northern part of Belgium. This enabled us to compare the four kinds of birth settings determined by country (Belgium versus the Netherlands) and place of birth (home versus hospital).

### Procedure

Women were asked by their midwife (primary care) or their obstetrician (secondary care) to participate in the research project. In both Belgium and the Netherlands, participants had to speak and understand Dutch and had to be 18 years or older. Questionnaires were returned to the midwife or obstetrician in a closed envelope. For practical reasons the Dutch women with a home birth sent the questionnaires straight to the researcher by mail. Women who delivered in a hospital for the most part completed the second questionnaire during their postpartum stay on the maternity ward. Women with a short stay, however, responded by direct mail instead. A written informed consent has been asked of all respondents, without connection to the questionnaire. No other personally identifiable data was collected. Hence, anonymity was ensured. The Committee for Ethics of the Ghent University Hospital has approved the study.

We had little control over the inclusion process and therefore the response rate, because obstetricians and midwives recruited respondents. Although we asked that women who refused to participate be registered, not everybody did this systematically. In consequence we can only give a minimum and maximum estimation of the response rate. During the preparation of the study, midwives and obstetricians gave an estimation of the eligible women within the three-month time frame provided. This estimation is reflected in the number of provided questionnaires. The response rate is calculated by dividing the number of respondents by the number of provided questionnaires. The estimations ranged between 19% and 68% for the hospitals, and between 38% and 100% for the midwifery practices. Midwives and physicians may have been selective about who they asked to participate in the study.

## Results

Within the first two weeks after delivery, 605 women, of which 261 are Belgian and 344 are Dutch, filled out a questionnaire. In our analysis we focus on this follow-up data. The number of cases in the analysis was reduced to 563, because 19 women left the planned place of delivery blank. Due to missing information on the control variables method of delivery and level of education, another 23 women dropped out of the analysis.

### Descriptives

In our sample the age of women ranges between 19 and 44 years, with a mean of 31 years. Dutch women were on the average slightly older at first birth (29.7 versus 28.05 years). Those having their first baby made up 45.8% of the population, and 98.7% were married or living as married. In the Belgian group there were 10% more primigravids. More Belgian (76.1%) than Dutch (40.8%) women completed higher education, and 85.9% of all women were employed, with 84.7% in Belgium and 86.8% in the Netherlands. Of our respondents, 22.5% had a medically assisted delivery (forceps, vacuum extraction or C-section), with 20.8% in Belgium, compared to 23.9% in the Netherlands. A home birth was planned for 37.0% of our respondents. In the Belgian region planned home births represent 24.0% in our sample, compared to 48.0% in the Netherlands. We remind the reader of the oversampling of home births (Table 1).

**Table 1:** Socio-demographic variables of Belgian and Dutch respondents

	***Total***		***Belgium***		***the Netherlands***		
	***n***	***Mean or %***	***n***	***Mean or %***	***n***	***Mean or %***	***p***

Higher education	329	56.1	194	76.1	135	40.8	<.001
Married/cohabitating	596	98.7	257	98.4	339	98.8	.239
Primiparae	276	45.8	133	51.0	143	41.9	.006
Employed	517	85.9	221	84.7	296	86.8	.685
Medical intervention	133	22.5	54	20.8	79	23.9	.361
Planning for a home birth	301	37.0	90	24.0	211	48.0	<.001
Age at first birth	-	28.99	-	28.05	-	29.7	<.001
Age	-	31.21	-	30.41	-	31.87	<.001

In the Belgian sample 87 (34.3%) women wanted a home birth versus 167 (65.7%) a hospital birth (Table 2). In the Dutch sample 176 (63.5%) women intended to give birth at home versus 101 (36.5%) who preferred to be taken care of in hospital. In some cases things didn't work out as planned: 18 (7.1%) Belgian women planned a home delivery, but in fact gave birth in a hospital; 82 (29.6%) Dutch women planning for a home birth had a referral to the hospital (Table 2).

**Table 2:** Respondents according to planned and actual place of birth, country and parity

		Belgium		the Netherlands		Total	
		
Expected place	Actual place	primiparous	multiparous	primiparous	multiparous	primiparous	multiparous
Home	Home	21 (15.8%)	48 (37.8%)	26 (19.0%)	68 (34.9%)	47 (17.4%)	116 (36.0%)
Home	Hospital	13 (9.8%)	5 (3.9%)	51 (37.2%)	31 (15.9%)	64 (23.7%)	36 (11.2%)
Hospital	Hospital	95 (71.4%)	72 (56.7%)	39 (28.5%)	62 (31.8%)	134 (49.6%)	134 (41.6%)
Other referrals		4 (3.0%)	2 (1.6%)	21 (15.3)	34 (17.4)	25 (9.3)	36 (11.2)

Total		133 (100.0%)	127 (100.0%)	137 (100.0%)	195 (100.0%)	270 (100.0%)	322 (100.0%)

The mean of the total Mackey Childbirth Satisfaction Rating Scale is 4.18 (st. dev. = 0.53), which is equal to the mean (4.18) reported in the study of Goodman et al. [16], although we omitted the physician-related items. The means of the subdimensions compare as follows (Goodman et al. versus our means): general: 4.2 versus 4.3; self: 3.8 versus 3.8; baby: 4.1 versus 4.4; midwife: 4.5 versus 4.5; partner: 4.3 versus 4.7. In both countries women were the least satisfied with self-related aspects of birth, with 48.1% on the Belgian side and 30.4% on the Dutch side. In Belgium support of the midwife accounted for the largest percentage of satisfied women (85.5%), and in the Netherlands support of the partner (69.0%). Note that in both Belgium and the Netherlands more women reported being (very) satisfied with the support and skills of the midwife (85.5% and 66.1% respectively) than with the doctor (71.7% and 47.9%) (Table 3).

**Table 3:** Childbirth satisfaction levels

	***Total***			***Belgium***			***the Netherlands***			
	***%***	***Mean***^1^	***St.dev*.**	***%***	***Mean***^1^	***St.dev*.**	***%***	***Mean***^1^	***St.dev*.**	***p***

Total	66.3	4.18	.53	78.2	4.35	.46	57.1	4.06	.56	<.001
General	47.3	4.03	.72	56.1	4.15	.67	40.8	3.93	.74	<.001
Self	38.0	3.81	.71	48.1	3.99	.66	30.4	3.67	.72	<.001
Baby	69.7	4.39	.77	75.1	4.49	.76	65.6	4.32	.77	<.008
Midwife	74.6	4.46	.66	85.5	4.62	.55	66.1	4.34	.72	<.001
Physician	61.9	4.20	.75	71.7	4.36	.71	47.9	4.06	.75	<.001
Partner	74.6	4.66	.53	81.5	4.73	.46	69.0	4.59	.57	<.001

^1 ^minimum = 1 and maximum = 5

### Linear regression model

We estimated a regression model for five subdimensions (general, self, baby, midwife, partner) and total satisfaction with childbirth. The model consists of two independent variables: the first is actual versus preferred or planned place of birth, which is a categorical variable with four groups: women intending to give birth at home who did, women planning to give birth at the hospital who did (reference group), women who were referred from home to hospital, and women who gave birth at another, unexpected place (e.g., home instead of hospital). The second independent variable is country, Belgium versus the Netherlands (reference group). Age, parity, education and method of delivery are controlled for. Results are shown in Table 4. Note that the reference groups are the Dutch and the women who wanted to give birth at hospital and did.

**Table 4:** Coefficients for satisfaction with childbirth (adjusted for method of delivery, parity, education and age) (N = 563)

		TOTAL^1^			SUBDIMENSIONS														
					GENERAL			SELF			BABY			MIDWIFE			PARTNER		

		B	s.e.	*p*	B	s.e.	*p*	B	s.e.	*p*	B	s.e.	*p*	B	s.e.	*p*	B	s.e.	*p*

(Constant)		3.757	.165	<.001	3.559	.235	<.001	3.244	.228	<.001	4.026	.242	<.001	4.185	.223	<.001	4.850	.190	<.001
Country		.309	.047	**<.001**	.123	.070	.082	.287	.068	**<.001**	.183	.069	**.008**	.339	.063	**<.001**	.161	.054	**.003**
Place of birth	Hospital *(reference group)*	-	-	-	-	-	-	-	-	-	-	-	-	-	-	-	-	-	-
	Home	.427	.051	**<.001**	.296	.073	**<.001**	.546	.071	**<.001**	.223	.076	**.003**	.444	.069	**<.001**	.154	.058	**.009**
	Hospital after referral	-.074	.059	.217	-.327	.095	**.001**	-.166	.092	.072	-.091	.087	.298	-.058	.081	.473	.055	.068	.422
	Other referrals	.034	.071	.632	-.068	.102	.504	-.001	.099	.990	-.059	.104	.569	.151	.096	.116	.080	.083	.334
	Country*hospital after referral	-	-	-	.460	.189	**.015**	.374	.182	**.041**	-	-	-	-	-	-	-	-	-
	Adjusted R^2^	.24			.16			.20			.23			.15			.04		

^1 ^Total over all subdimensions, except satisfaction with the physician.

Legend

Hospital: women who expected to give birth at hospital, and did.

Home : women who expected to give birth at home, and did.

Hospital after referral: women who expected to give birth at home, but were referred to the hospital.

Other referrals: women with other discrepancies between expected and actual place of birth.

The two countries, Belgium and the Netherlands, are characterised by diverging satisfaction scores. Belgian women are more satisfied with childbirth in total (B = 0.31; s.e. = 0.05; p < 0.001) and for all but one subdimension (self: B = 0.29; s.e. = 0.07; p < 0.001; baby: B = 0.18; s.e. = 0.07; p = 0.008; midwife: B = 0.34; s.e. = 0.06; p < 0.001; partner: B = 0.16; s.e. = 0.05; p = 0.003). Note that the Belgian women have an advantage over the Dutch especially in terms of the midwife's support.

Regarding place of birth, we compared women who intended to give birth at home and did, women who planned a home birth but were referred to the hospital, and women with other discrepancies between plan and reality, with women who intended to give birth in hospital and did. When comparing women who gave birth at the place they intended to, it is clear that home births are consistently (total: B = 0.43; s.e. = 0.05; p < 0.001; general: B = 0.30; s.e. = 0.07; p < 0.001; self: B = 0.55; s.e. = 0.07; p < 0.001; baby: B = 0.22; s.e. = 0.08; p = 0.003; midwife: B = 0.44; s.e. = 0.07; p < 0.001; partner: B = 0.15; s.e. = 0.06; p = 0.009) more satisfying than hospital births, especially regarding the self- and midwife-related aspects. Women who have been referred from home to the hospital report lower general satisfaction scores (B = -0.33; s.e. = 0.10; p = 0.001) compared to women who planned and had a hospital birth. However a referral from home to hospital is inconsequential in terms of the other subdimensions of satisfaction (self: B = -0.17; s.e. = 0.09; p = 0.072; baby: B = -0.09; s.e. = 0.09; p = 0.298; midwife: B = -0.06; s.e. = 0.08; p = 0.473; partner: B = 0.06; s.e. = 0.07; p = 0.422). The satisfaction of women who gave birth at other, unplanned for places, (e.g., home instead of hospital or hospital instead of short stay) did not diverge from that of women who intended to give birth at hospital and did (total: B = 0.03; s.e. = 0.07; p = 0.63; general: B = -0.07; s.e. = 0.10; p = 0.504; self: B = -0.001; s.e. = 0.10; p = 0.990; baby: B = -0.06; s.e. = 0.10; p = 0.569; midwife: B = -0.15; s.e. = 0.10; p = 0.116; partner: B = 0.08; s.e. = 0.08; p = 0.334).

To test whether place of birth is associated differently with satisfaction in the two countries, we included three interaction terms, one for each dummy, in our analysis but retained only the significant term, which is "hospital after referral*country". The benefits of a home birth are equal in Belgium and the Netherlands (in total and for every subdimension), but the disadvantage of being referred to the hospital when a home birth was expected is smaller in Belgium. Regarding general satisfaction (B_country*place _= 0.46; s.e. = 0.19; p = 0.015) and satisfaction with self-related aspects (B_country*place _= 0.37; s.e. = 0.18; p = 0.041) of birth, Belgian referred women are more satisfied than Dutch women. The coefficients show that when comparing women who had a hospital birth they had planned for with women who had a hospital birth after referral, Dutch referred women are the least satisfied with their birth experience, while Belgian referred women are the most satisfied. Women who had a hospital birth they had planned for, fell somewhere in between and their scores did not differ in Belgium and the Netherlands. In other words, Belgian women who have been referred to the hospital during pregnancy or labour have higher satisfaction scores than Belgian women who planned to give birth in hospital and did. The reverse is true in the Netherlands.

Regarding the control variables (no table), it is clear that multiparous women are generally more satisfied about the birth experience (general: B = 0.20; s.e. = 0.06; p = 0.002) and about the baby-related aspects (baby: B = 0.18; s.e. = 0.06; p = 0.005), but less satisfied about the partners' support (B = -0.12; s.e. = 0.05; p = 0.017). Method of delivery is important for most of the dimensions of satisfaction with childbirth (total: B = -0.18; s.e. = 0.05; p = 0.001; general: B = -0.19; s.e. = 0.07; p = 0.011; baby: B = -0.66; s.e. = 0.08; p < 0.001; midwife: B = -0.14; s.e. = 0.07; p = 0.047), even after introducing place of birth, except for satisfaction with self- (B = -0.07; s.e. = 0.07; p = 0.342) and partner (B = -0.07; s.e. = 0.06; p < 0.289) related aspects. Medical interventions during birth are especially relevant for satisfaction concerning the baby, which is not surprising.

Our results show that place of birth, more specifically being able to give birth at an expected place, determines how mothers evaluate the birth experience. Moreover this feature operates in a different way in Belgium and the Netherlands. The finding that Belgian referred women are more satisfied than Belgian women who planned to give birth at hospital and did, the opposite of which is true for the Dutch, is most remarkable. Place of birth, one of the central differences between the Belgian and Dutch maternity care system, explains part of the diverging satisfaction scores of Dutch and Belgian women.

## Discussion

Advocates of hospital births often use referrals as an argument against home birthing, assuming that it is a disappointing experience [3] resulting in a lowered satisfaction with childbirth. We have addressed the impact on postpartum satisfaction of being referred in both a maternity care system favouring home births (the Netherlands), and one that predominantly labels home births as risky and advocates hospital births (Belgium). Because of the lack of continuity of care in case of referral, we expected that a referral would have the greatest negative effect in Belgium.

Before further discussing the findings, we want to briefly list some of the shortcomings and merits of the study. First, our data consists of a convenience sample limited to two comparable Belgian and Dutch cities. This makes generalisability to the Belgian and the Dutch population uncertain. Second, there is no ideal time to measure satisfaction. In our study, respondents filled out questionnaires within two weeks after childbirth for practical reasons. This close to the birthing experience, women might have answered less critically than they would have later on [20]. However, the two-week time frame applied to all respondents and therefore does not affect the differences between the groups compared. Third, place of measuring satisfaction might be problematic, because it differs for women who had a home birth and women who delivered in hospital. The former answered the questionnaires at home, the latter in hospital. Studies have shown that women answering at home are more critical compared with women who fill in the questionnaire in hospital, due to loyalty to the institution [21]. We find that women giving birth at home are more satisfied than women giving birth in hospital. If the former answered the questionnaires more critically, this finding is even more salient. Fourth, comparability of the Dutch and Belgian sample can be questioned: Belgian women are on the average more highly educated, younger at first birth and more likely to give birth for the first time in comparison to the Dutch. The higher education of the Belgian sample can be explained by the oversampling of home births, since in Belgium women preferring a home birth are on the average more highly educated [10]. In the Netherlands women are on the average older at first birth in comparison to Belgium and the rest of Europe [22]. Age and education are controlled for in the analysis. Next, the response rate of some hospitals was rather low. We examined the impact by running the analysis with and without the respondents from these hospitals. By eliminating the respondents who gave birth in a low-response hospital, the total number of respondents in the restricted sample decreased to 466, compared to 563 in the full sample. In general the main country effect increased a little, but there were no substantial changes. Finally, we did not distinguish between women who have been referred during the last eight to ten weeks of pregnancy and women with an intrapartum referral. The group of women who have been referred (N = 100) consists half of women being referred during pregnancy and half of women referred during labour; 82 are Dutch and 18 Belgian. These small numbers make inclusion in a regression analysis inappropriate. In addition, we did not find significant differences in satisfaction between women with a referral during pregnancy and those with a referral during labour. Moreover, we were especially interested in the effect of the discrepancy between expected and actual place of birth on satisfaction, no matter when this discrepancy occurred.

The merits of this research lie in the cross-national comparison and the conceptualisation of satisfaction with childbirth. Cross-national comparison in midwifery literature is often of a qualitative rather than a quantitative design. Moreover cultural differences are often the major focus. We concentrate on structural differences between birth practices and maternity care systems. Referrals are a typically Dutch phenomenon, since the Netherlands is characterised by a unique system encouraging home births. Research on the impact of a referral to specialist care is rare and often limited to the Dutch population, since home births are a rare phenomenon outside of the Netherlands. In addition, the analysis for each subdimension (general, self, baby, midwife, partner) separately, shows variation in outcomes across subdimensions. This finding affirms the importance of narrowing down the construct of satisfaction with childbirth to its subdimensions.

## Conclusion

Our main finding is the negative effect of being referred to hospital when a home birth was planned on (1) satisfaction in general and (2) self-related aspects of birth. Since self-related items in the questionnaire focus on personal control, this could indicate a sense of a lack of control in cases of referral. Note that the negative effect of being referred does not affect satisfaction related to the baby, the midwife or the partner. Regarding the satisfaction with midwife's support, the conclusions of Wiegers et al. [3] are affirmed: Dutch women who intended to give birth at home but were referred to hospital were as positive about the attendance of the midwife as the women who had the hospital birth they planned for.

The disadvantages of being referred are especially true in the Netherlands. An explanation for the differing impact of being referred to specialist care in Belgium versus the Netherlands could be sought in the diverging quality of care after referral. However, we have two reasons to believe this is not the case. First, a referral does not affect satisfaction with birth attendants, but is limited to general and self-related satisfaction. Second, if differences in quality of care occur, we would expect Dutch maternity care to do a better job than the Belgian system, which does not provide procedures to take care of referred women. A reviewer pointed out that in the Netherlands community midwives can accompany their client to the hospital, although in practice continuity of care depends on the stage of labour and on the midwife. A Dutch referral often ends up in a short stay, which is still closer to the ideal type of the midwifery/social model than the Belgian hospital births. In other words, continuity of care is more likely in the Netherlands, in practice as well as ideology. Thus far, there is no reason to believe Dutch women receive poorer quality hospital care than Belgian women after transfer.

The reference group theory provides a post hoc explanation, taking the subjective situation of Belgian and Dutch women into account. Merton [23] introduced the concept of relative deprivation to explain feelings of dissatisfaction in cases where the objective situation does not seem to account for such feelings. In the Netherlands home births are the point of reference for most women, since 70% [6] start antenatal care with primary caregivers, corresponding with the national strategy encouraging home births. In other words (most) Dutch women believe that home births are the most desirable. In consequence, in cases of referral to specialist care, they will feel relatively deprived in comparison with the reference group of women with positive home birth experiences. Although we make the assumption that home births are preferred, it is in fact unclear to what extent Dutch women personally prefer home births or are merely constrained by the system. In contrast, we know that Belgian women who choose a home birth have an actual preference for giving birth at home. These women consciously question the dominant bio-medical approach and encounter disapproval from family or friends. The Belgian women planning for a home birth will never find a consensus about home birthing in their direct social network. In case of conflicting expectations in the direct social environment, Merton [23] theorises that individuals do not take significant others as point of reference, but rely on the norms and expectations of the broader societal context. Consequently, Belgian women planning for a home birth will not experience relative deprivation; hence their satisfaction scores will not drop in the same way as the Dutch satisfaction scores. Being referred to a hospital in Belgium is being obliged to conform to the normative way of giving birth.

In sum, home births lead to higher satisfaction, but once a referral to the hospital is necessary satisfaction drops and ends up lower than for hospital births planned in advance. At least this is true for the Netherlands. In Belgium referred women are more satisfied than women who had the hospital birth they planned, but less satisfied than women who had the home birth they wished for. We need to understand more about referral processes in the national context of the organisation of maternity care and how women experience them.

## Competing interests

The author(s) declare that they have no competing interests.

## Authors' contributions

WC designed the study, organised the data collection, and was responsible for the analysis of the data. She drafted the manuscript and revised it. This was reported back to AG and PB.

PB formulated the research question and contributed to the conception of the study. AG contributed to the analysis of the data. AG and PB critically reviewed draft versions of the manuscript. All authors contributed to the development of this manuscript. All authors read and approved the final manuscript.

## Pre-publication history

The pre-publication history for this paper can be accessed here:



## References

[B1] Christiaens W, Bracke P (2007). Place of birth and satisfaction with childbirth in Belgium and the Netherlands. Midwifery doi:10 1016/j midw 2007 02 001.

[B2] De Vries R (2001). Midwifery in the Netherlands: vestige or vanguard?. Medical Anthropology.

[B3] Wiegers TA, van der Zee J, Keirse MJNC (1998). Transfer from home to hospital: what is its effect on the experience of childbirth?. Birth-Issues in Perinatal Care.

[B4] Wiegers TA, van der Zee J, Keirse MJNC (1998). Maternity care in the Netherlands: the changing home birth rate. Birth-Issues in Perinatal Care.

[B5] (2007). http://europe.obgyn.net.

[B6] Anthony S, Amelink-verburg MP, Jacobusse GW, van der Pal-de Bruin KM (2005). De thuisbevalling in Nederland 1995-2002. Rapportage over de jaren 2001-2002 (Home births in the Netherlands 1995-2002. Report for the years 2001-2002).

[B7] Reuwer PJHM, Bruinse JW (2002). Preventive support of labour, een uitdaging voor verloskundigen, gynaecologen en beleidsmakers (Preventive support of labour, a challenge to midwives, obstetricians and policymakers).

[B8] van Teijlingen E (2005). A critical analysis of the medical model as used in the study of pregnancy and childbirth. Sociological Research Online.

[B9] Davis-Floyd R (2001). The technocratic, humanistic, and holistic paradigms of childbirth. International Journal of Gynecology & Obstetrics.

[B10] Gilleir C (2007). Thuis bevallen in Vlaanderen: een kwestie van reflexiviteit (Home birth in Flanders: a matter of reflexivity). Tijdschrift voor Sociologie.

[B11] Cammu H, Martens G, De Coen K, Van Mol C, Defoort P (2005). Perinatale activiteiten in Vlaanderen 2005 (Perinatal activities in Flanders 2005).

[B12] Green JM, Renfrew MJ, Curtis PA (2000). Continuity of carer: what matters to women? A review of the evidence.. Midwifery.

[B13] Staniszewska S, Ahmed L (1999). The concepts of expectation and satisfaction: do they capture the way patients evaluate their care?. Journal of Advanced Nursing.

[B14] Cleary PD, McNeil BJ (1988). Patient satisfaction as an indicator of quality care. Inquiry-the Journal of Health Care Organization Provision and Financing.

[B15] Sitzia J, Wood N (1997). Patient satisfaction: a review of issues and concepts. Social Science & Medicine.

[B16] Goodman P, Mackey MC, Tavakoli AS (2004). Factors related to childbirth satisfaction. Journal of Advanced Nursing.

[B17] Green JM (1993). Expectations and experiences of pain in labor--Findings from a large prospective study. Birth-Issues in Perinatal Care.

[B18] Slade P, Macpherson SA, Hume A, Maresh M (1993). Expectations, experiences and satisfaction with labor. British Journal of Clinical Psychology.

[B19] Bramadat IJ, Driedger M (1993). Satisfaction with childbirth--Theories and methods of measurement. Birth-Issues in Perinatal Care.

[B20] van Teijlingen E, Hundley V, Rennie AM, Graham W, Fitzmaurice A (2003). Maternity satisfaction studies and their limitations: "What is, must still be best". Birth-Issues in Perinatal Care.

[B21] Lumley J (1985). Assessing Satisfaction with Childbirth. Birth-Issues in Perinatal Care.

[B22] Beets G (2004). De timing van het eerste kind: een overzicht (Timing of the first child: an overview). Bevolking en Gezin.

[B23] Merton MK (1968). Social theory and social structure.

